# Delayed Meniscus Repair Lowers the Functional Outcome of Primary ACL Reconstruction

**DOI:** 10.3390/jcm13051325

**Published:** 2024-02-26

**Authors:** Patrick Sadoghi, Harald K. Widhalm, Martin F. Fischmeister, Lukas Leitner, Andreas Leithner, Stefan F. Fischerauer

**Affiliations:** 1 Department of Orthopaedics and Trauma, Medical University of Graz, Auenbruggerplatz 5, 8036 Graz, Austria; patrick.sadoghi@medunigraz.at (P.S.); lukas.leitner@medunigraz.at (L.L.); andreas.leithner@medunigraz.at (A.L.); 2 Clinical Division of Traumatology, Department of Orthopedics and Traumatology, Medical University of Vienna, 1090 Vienna, Austria; harald.widhalm@meduniwien.ac.at; 3 AUVA Traumacenter Unfallkrankenhaus Linz, 4017 Linz, Austria; martin@fischmeister.info

**Keywords:** anterior cruciate ligament reconstruction, meniscus, early versus delayed surgery

## Abstract

Background: Our purpose was to evaluate whether the time of intervention and the type of meniscus surgery (repair vs. partial meniscectomy) play a role in managing anterior cruciate ligament (ACL) reconstructions with concurrent meniscus pathologies. Methods: We performed a prospective cohort study which differentiated between early and late ACL reconstructions with a cut-off at 3 months. Patients were re-evaluated after 2 years. Results: Thirty-nine patients received an operation between 2–12 weeks after the injury, and thirty patients received the surgery between 13–28 weeks after trauma. The strongest negative predictive factor of the International Knee Documentation Committee subjective knee form in a hierarchical regression model was older age (ß = −0.49 per year; 95% CI [−0.91; −0.07]; *p* = 0.022; partial R^2^ = 0.08)). The strongest positive predictive factor was a higher preoperative Tegner score (ß = 3.6; 95% CI [0.13; 7.1]; *p* = 0.042; partial R^2^ = 0.07) and an interaction between meniscus repair surgery and the time of intervention (ß = 27; 95% CI [1.6; 52]; *p* = 0.037; partial R^2^ = 0.07), revealing a clinical meaningful difference as to whether meniscus repairs were performed within 12 weeks after trauma or were delayed. There was no difference whether partial meniscectomy was performed early or delayed. Conclusions: Surgical timing plays a crucial role when surgeons opt for a meniscus repair rather than for a meniscectomy.

## 1. Introduction

During sports injuries, the prevalence of anterior cruciate ligament (ACL) tears that are accompanied by meniscal injuries has been reported in 44–63% of cases [1,2,3]. Successively, the rate of concomitant meniscal procedures during ACL reconstructions has increased from the years 2000 to 2016 by 49–60% [4]. Saving the meniscus via repair techniques is the preferred method as it is well established that meniscectomies lead to the development of premature osteoarthritis [5,6]. The importance of the meniscus on the joint maintenance and cartilage longevity is due to its unique function as a shock absorber [7], a load distributor minimizing excessive contact pressure [8], and a secondary stabiliser of the knee [9]. Unfortunately, the meniscus possesses poor regenerative potential due to its intra-articular location and relative avascularity [10]. It would be a preferable setting to repair all meniscus tears; however, overall long-term failure rates remain high, with around 19% to 25% in average [11,12]. About two-third of failures occur within the first two years after surgery [12]. Potential factors associated with failed repairs are the type of tear, the zone of the injury, the age of the patient, and the chronicity of the tear [13,14]. ACL-deficient knees can cause repeated micro trauma to the meniscus. Failure rates of meniscus repairs in ACL-deficient knees are in consequence found in 30–40% [15], whereas ACL reconstructions combined with meniscus repair provide success rates of around 75% [16,17,18]. It is therefore recommended to perform meniscus repairs in ACL-deficient knees simultaneously with ACL reconstructions. However, ACL reconstruction meniscectomy is still performed two or even three times more frequently than meniscus repairs [19].

Levy et al. and Frobell et al. have evaluated the appropriate timing for ACL reconstruction in the New England Journal of Medicine in 2011, describing the cut-off for early reconstruction versus delayed at around 3 months after the accident [20,21]. Some surgeons prefer early ACL reconstruction because restoring tibiofemoral stability in the early weeks after injury might prevent the risk of progressive meniscal tears or chondral damage [22,23]. In addition, early surgical intervention might facilitate the return to sports with all its socioeconomic consequences. In contrast to that, delayed ACL surgery might allow the patient to gain an optimal range of motion (ROM) with adequately recovered soft tissue, leading to less arthrofibrosis or wound complications [24]. Bierke et al. investigated the role of timing and meniscus sutures on the risk of arthrofibrosis in anatomical anterior cruciate ligament reconstruction [25]. The odds of receiving a subsequent arthroscopic arthrolysis was 4.4 times higher in patients operated within 6 weeks, and 3.4 times higher in patients who had undergone meniscus repair at the index surgery.

With respect to the adequate time of intervention, recommendations remain controversial, and data on long-term functional outcomes are rarely available. This is especially the case when taking concurrent meniscus surgeries into account. Hence, our aim was to assess the impact of two factors on patients who underwent ACL reconstruction and were followed up for two years. The factors in question were the timing of the operative intervention (within the first 12 weeks or after) and whether concurrent meniscus surgeries were performed. Our primary hypothesis was that there would be no significant difference in functional outcomes between those who had ACL reconstructions within three months of their injury and those who had it later. Additionally, we hypothesised that the functional outcome would not be affected by concurrent meniscus surgeries, whether they involved partial meniscectomy or repair.

## 2. Materials and Methods

### 2.1. Study Design and Setting

We performed a single surgeon prospective cohort at the AUVA, Unfallkrankenhaus in Linz, Austria between February 2006 and May 2008. Ethical approval was obtained from the responsible internal review board of the AUVA. All patients have given written informed consent to participate in the trial. The time of the surgical intervention was influenced by factors, such as the reference of the family practitioner, or the initial presentation of the patient. The patients were re-evaluated after a follow-up of 2 years.

### 2.2. Study Population

We included only unilateral ACL ruptures who fulfilled the criteria to undergo arthroscopically assisted ACL reconstruction. The exclusion criteria were defined as clinical situations that required complex surgical intervention (meniscus root tears, bucket-handle tears, ramp lesions), multi-ligament knee injuries (posterior cruciate ligament, posterolateral knee complex injuries, and lateral collateral ligament injuries), previous knee ligament surgeries, and fractures of the femur or tibia. Concurrent medial collateral ligament injuries (grade I–III) were treated conservatively with an orthosis for 6 weeks and were not excluded from the current study.

### 2.3. Surgical Techniques and Postoperative Care

Either a bone-patellar tendon-bone single-bundle (PTB-SB) or a four-tunnel semitendinosus-gracilis double-bundle (STG-DB) technique was performed for ACL reconstruction. The author M.F.F. operated on all patients as described previously [26,27]. The need for meniscal intervention was determined intraoperatively, guided by a multifactorial decision-making process. In instances of meniscal tears, the choice between meniscus repair techniques or partial meniscectomy was primarily contingent upon factors including tear stability, the surgeon’s proficiency, and the patient’s preferences. Tears characterised by stability, permitting secure suturing for alignment and healing, were considered appropriate indications for repair. Conversely, instances not meeting these criteria warranted a partial meniscectomy procedure. The same identical rehabilitation pathways were used for all patients according to the protocol of Shelbourne and Nitz [28], and all patients were sent to physiotherapy preoperatively before being scheduled for ACL reconstruction. There were no other variations concerning indications for the procedures and postsurgical care.

### 2.4. Outcome Measures

We assessed the demographic data (age, gender), injury characteristics (side, concomitant injuries), surgery characteristics (operating groups, meniscus repairs, surgery duration), the length of hospital stay, and pre- and postoperative functional assessments using the KT 1000 and patient-reported outcome measures (Tegner [29] and International Knee Documentation Committee (IKDC) subjective knee form [30]).

The Tegner scale was pre- and postoperatively assessed, providing a measure of working and sporting activity levels, ranging from 0 (“sick leave/disability”) to 10 (“participation in national and international elite competitive sports”).

The IKDC subjective knee form was measured postoperatively and provides a patient-reported measure of knee symptoms, function, and sports activities. Standardised scores range from 0 (lowest level of function or highest level of symptoms) to 100 (highest level of function and lowest level of symptoms).

### 2.5. Cohort Subgroups

In this study, two factors are of interest: the time of intervention and meniscus surgery. The primary cohorts that distinguish between early and late interventions are described in detail in Table 1. The subgroups of meniscus surgery were (i) none (reference), (ii) partial resection/debridement, and (iii) repair. Meniscus repairs were performed all arthroscopically with suturing techniques. The distribution of meniscus tear types within the subgroups are displayed in Table 2.

### 2.6. Statistical Analysis

We present patients’ characteristics by measures of central tendencies (e.g., proportion, mean, median) as appropriate. Bivariate comparisons between continuous and dichotomous were tested using an independent *t*-test for parametric data, and the two-sample Wilcoxon rank-sum test for nonparametric data. The paired *t*-test was used to evaluate pre- and postoperative outcome differences. Pearson’s Chi-square test and the Fisher exact test were used to test for relationships among two categorical factors. The Pearson’s correlation coefficient was determined to describe the relationship between two continuous variables. We further conducted a hierarchical linear regression to estimate whether long-term functional outcomes (IKDC) have an association with the time point of ACL and meniscus surgery above and beyond demographic and clinical variables that showed a significant association (*p* < 0.05), with IKDC scores in bivariate analysis. In Step 1, we included the relevant demographic variables with an association to the physical function in bivariate analysis (age, preoperative Tegner score). In Step 2, we added the factor time of surgery (early vs. delayed), and Step 3 specifies the operating procedure (ACL reconstruction technique, concurrent meniscus resection/repair). In Step 4, we finalised the regression model by adding an interaction term between meniscus surgery and the time of intervention. We report the coefficient of determination (R^2^) of the entire model, in each step as well as the partial R^2^ of each individual variable. According to Cohen, regression models in human sciences can be considered as very weak (<0.02), weak (0.02–0.13), moderate (0.13–0.26), or substantial (>0.26), respectively [31]. To assure that interpretations of the regression models are not biased, we performed post-estimation tests for heteroskedasticity (White’s test), skewness, kurtosis (both Cameron−Trivedi tests), and nonlinearity (Ramsey RESET test). We reported significance using an α level of 0.05. All analyses were performed using Stata/BE 17.0 (Stata Corp, College Station, TX, USA).

## 3. Results

**Figure 1:**
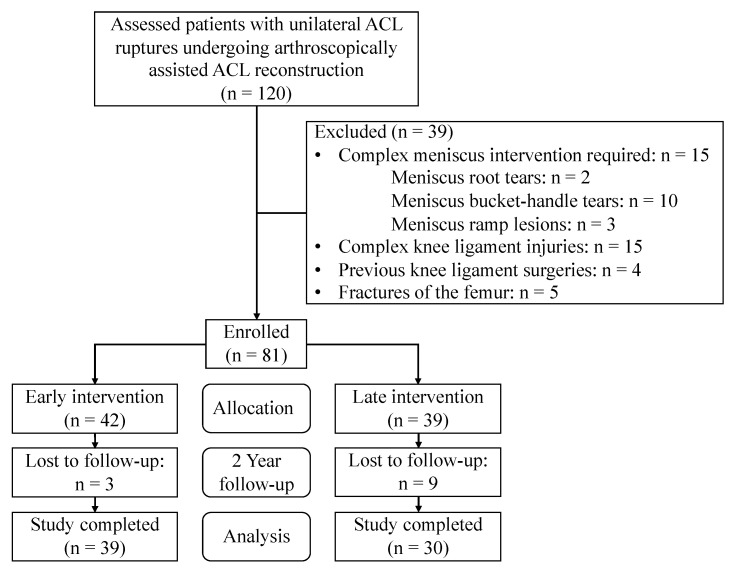
Flow diagram of patients allocated to the study.

Of the initially assessed 120 patients, 39 were excluded, and 81 were enrolled and allocated to the intervention groups (Figure 1). Twelve patients were lost to follow-up, and sixty-nine patients remained eligible for study analysis. The descriptive analysis of the study population, as well as the subgroup analysis of early and late interventions are displayed in Table 1. The study population was on average 35 ± 11 years old. Of these, thirty-five patients (51%) suffered a concurrent meniscus injury. The meniscus pathologies were horizontal tears in 17 patients (49%), flap tears in 13 patients (37%), and radial tears in 5 patients (14%) (Table 2). One patient injured both the medial and lateral meniscus. The preoperative Tegner score ranged from 0 to 5 (median 1 [IQR: 1–2]).

We did neither observe any group differences, nor any differences in the mid-term functional outcome with regard to the used ACL reconstruction technique. Twenty-four patients received concomitant partial meniscectomy with the ACL reconstruction, and eleven patients underwent meniscus repair (Table 2). Statistically, there was no relationship between the meniscus pathology and the performed surgery technique (meniscectomy vs. repair).

### 3.1. Evaluation of Early versus Late ACL Intervention

A total of 39 patients (57%) were operated 2–12 weeks after injury, and 30 patients (43%) received their surgery between 13 and 28 weeks after injury. The bivariate analysis showed that patients in the early intervention group had an overall higher postoperative IKDC score (ß = 9.6; 95% CI [−19; −0.23]; *p* = 0.045; independent *t*-test) and a higher postoperative Tegner score (*p* = 0.027; independent *t*-test) at a mean follow-up period of 2.8 ± 0.65 years. All patients showed improvement between the preoperative and postoperative Tegner scores (ß = 3.8; 95% CI −4.2; −3.4]; *p* = <0.001; paired *t*-test), but the amount of improvement was not significantly associated with the intervention group (ß = 0.59; 95% CI [−1.4; 0.20]; *p* = 0.140; independent *t*-test). Bivariate analysis confirmed that there was a significant association between IKDC outcomes and preoperative Tegner scores (r = 0.31; *p* = 0.009; Pearson’s correlation coefficient). The only further factor that showed an association with the IKDC scores in bivariate analysis was age (r = −0.24; *p* = 0.049; Pearson’s correlation coefficient). We did not find an association between the timing of the intervention and the performed meniscus surgeries with the KT-1000 results.

### 3.2. The Factor-Specific Evaluation of the Time of Intervention and Meniscus Surgery

To analyse the factor-specific influence on the variations in IKDC scores, we performed a hierarchical regression analysis. We stepwise included all scientific relevant factors and controlled for potential confounders, such as age and preoperative Tegner scores (Table 3). In Step 1, demographics were included into the model, and the preoperative Tegner score, except for the age, showed to have a significant association with the postoperative IKDC scores. At this stage, the model was able to explain a moderate number of variations in the IKDC score (R^2^ = 0.14), showing that age (partial R^2^ = 0.05) and preoperative Tegner scores (partial R^2^ = 0.09) were strong contributors. By adding the time of intervention (early vs. late ACL reconstruction) into the model at Step 2, the explanation capacity of the model increased by ΔR^2^ = 0.04. A further 1% in explaining the variations of IKDC was achieved in Step 3 by adding concomitant surgery specifics whether (i) no (reference), (ii) a partial resection, or (iii) a repair surgery of the meniscus was performed. None of these factors appeared to have a significant relation to the functional outcome so far in the model. Finally, in Step 4, the interaction term between the meniscus surgery and the time of intervention was added to the model. The overall factor of early intervention remained insignificant, as did the interaction between whether a meniscus pathology was partially resected at an early or late intervention. However,, we found a significant interaction between the time of intervention and when a meniscus repair was performed (ß = 27; 95% CI [1.6; 52]; *p* = 0.037). This interaction factor accounted for 7% of the variation in the IKDC score. The final model was able to explain 25% of the variations in postoperative IKDC scores. In the final regression model predicting the outcome of IKDC measures, the strongest negative predictive factor was older age (ß = −0.49 per year; 95% CI [−0.91; −0.07]; *p* = 0.022; R^2^ = 0.08), and the strongest positive predictive factor was a preoperative higher Tegner score (ß = 3.6; 95% CI [0.13; 7.1]; *p* = 0.042; R^2^ = 0.07) and the interaction between meniscus repair surgery and the time of intervention (ß = 27; 95% CI [1.6; 52]; *p* = 0.037; R^2^ = 0.07). All other factors did not show a significant association with the postoperative physical function. Post-estimation tests confirmed that no violations evaluating our regression model were present (heteroskedasticity (Chi^2^ = 25; *p* = 0.203), skewness (Chi^2^ = 5.3; *p* = 0.62), kurtosis (Chi^2^ = 2.2; *p* = 0.14), and nonlinearity (F (6, 55)) = 0.99; *p* = 0.442). The main finding in the postoperative outcome between the subgroups early and late meniscus repair revealed a post hoc power of >0.99 as the observed effect size was very large (d = 2.2).

## 4. Discussion

In our study on patients receiving an ACL reconstruction, we observed that a concomitant meniscus repair at an early stage achieved significantly higher functional outcomes when compared to late interventions. We further found that concomitant partial meniscectomies did not alter the mid-term functional outcome independently of the time of intervention. Our final model was able to explain a moderate-to-substantial number of variations (25%) in the postoperative IKDC scores.

The definition of an early intervention after an ACL injury was described as prior to 12 weeks by Maffulli et al. [32]. Significantly, there is a controversy regarding the time of the cut-off regarding early versus delayed ACL reconstruction. A meta-analysis by Shen et al. evaluated 11 randomised controlled trials, including 972 patients in total, using the definition of early ACL reconstruction within 10 weeks. They found that early reconstruction was not associated with improved functional outcomes, nor with fewer complications [33]. Different time periods were defined by Evans et al., with early reconstructions within 3 weeks, and delayed reconstructions after 6 weeks [34], stating that there is still debate regarding the optimal timing. In the study of Bierke et al., there was no significant difference found in the functional outcome after ACL reconstruction performed 12 months postoperatively when comparing early (under 6 weeks) versus late (beyond 6 weeks) interventions [25]. Our study results confirm that if the time point of solely ACL reconstruction is evaluated, the functional outcome does not change whether the ACL surgery was performed at an early or later stage. This was also confirmed within objective measures using the KT-1000. However, we observed a significant change of the outcome when the time of intervention was evaluated with respect to an accompanied meniscus repair. The prevalence of medial meniscus tears in our study population was 36%. Similar percentages were reported in a study by Kimura et al. [35]. The prevalence of combined ACL and lateral meniscus tears in the study by Kimura et al. was 56% [35]. In a meta-analysis by Sarraj et al., meniscal resection demonstrated better symptoms at a 2-year follow-up when compared to patients with ACLR combined with meniscal repair. Taking the time of intervention into account, our study results showed that the mid-term functional outcome after partial meniscectomy was independent from the time of intervention. Contrastingly, we observed a tremendous difference as to whether a meniscus repair was performed at an early or late intervention time. Higher failure rates at late interventions have also been reported by Karuppiah et al. [36]. Avascularity and poor opposing margins of the meniscus edges may be reasons for the poor healing potential of chronic tears [37]. Higher success rates of early meniscal repair align with the results of Uzun et al. [38]. In that study, all failed cases of meniscal repair occurred in the group that was operated on 8 weeks after the injury. In our study, the functional differences between early and late repairs were 27 points in the IKDC score (*p* = 0.043). This substantial difference did thereby exceed the minimal clinically important difference (MCID) threshold of 18.6 points for IKDC scores after ACL reconstruction, as reported by Beletsky et al. [39]. The time of intervention when the meniscus was repaired was also one of the main contributing factors to the overall model in explaining the functional outcome. The drawback of delayed surgery might specifically be predominant when a surgery involves the necessity of a meniscus repair. To prevent cartilage damage, muscular atrophy, deconditioning, and prolonged rehabilitation [40,41,42,43], an early intervention of ACL-deficient knees with repairable meniscus pathologies can be recommended. Regarding our findings of negative association between older ages and IKDC outcomes, we postulate that advanced age may be correlated with diminished tissue healing capacity, reduced physiological resilience, and potentially slower rehabilitation progress. Conversely, the positive association between a higher preoperative Tegner score and an improved IKDC outcome aligns with the notion that individuals with a higher baseline activity level may exhibit greater motivation, adherence to rehabilitation protocols, and better overall functional outcomes. The acknowledgment of older age as a negative predictor and a higher preoperative Tegner score as a positive predictor holds practical implications for clinicians. Recognizing age-related challenges and tailoring rehabilitation strategies accordingly may be crucial for optimizing outcomes. Similarly, the preoperative assessments of activity levels can guide clinicians in devising personalised rehabilitation plans in order to enhance functional recovery.

### Limitations

We were able to describe a moderate-to-substantial amount of the variation in the functional outcome of IKDC scores; however, some important factors, such as psychosocial involvement or any high-grade chondral defects that have been named the most consistent and potentially largest negative effect on long-term patient-reported outcomes [44], have not been regarded in this study. Although the IKDC score is divided into both an objective and subjective part, we lack the objective methods to evaluate the rotational laxity and quantify anterior knee pain, which is subjectively answered by the patients. The Tegner scale, commonly used in orthopaedics to classify activity levels, provides a general overview, but lacks specificity in detailing the nature, intensity, and duration of activities. This limitation is particularly notable in the evaluation of outcomes following ACL reconstruction and meniscus surgery. To address this limitation, further studies may consider supplementing the Tegner scale with additional instruments or questionnaires that delve into the specific nature of activities, their intensity, and the duration of engagement. We also want to underline the limitation of timing, specifically regarding the cut-off period for early versus delayed reconstruction, as this was set differently across various publications, which limits the ability to meta-analyse the datasets accordingly. One notable limitation of our study pertains to the inclusion of meniscal tears. While we excluded ramp lesions, bucket-handle lesions, and root tears due to their unique characteristics, we did not identify substantial differences in outcomes or reinjury risks among the remaining tear types. Additionally, our study’s sample size may not have provided sufficient statistical power to detect any nuanced differences between tear-type subgroups. Furthermore, the decision to repair or resect was influenced by multiple factors, including tear stability, surgeon expertise, and patient preferences, which may have introduced variability into the analysis. Despite the small subgroups of patients receiving a concomitant meniscus repair in both early and late intervention groups, the difference found in the functional outcome was large enough power to allow observation of the mentioned effect. However, this research may lack a generalizability to the larger population, due to the very limited sample size. These facts underscore the need for larger, more specific studies which are dedicated to exploring the outcomes within distinct meniscal tear subtypes, which could then provide deeper insights into the complexities of meniscal interventions.

## 5. Conclusions

The functional mid-term outcome after ACL surgery and concomitant meniscus injuries was significantly higher when meniscus repairs were performed at an early time of intervention. We recommend that patients with a combination of ACL reconstruction and repairable meniscus pathology are treated within 12 weeks of the initial injury.

## Figures and Tables

**Table 1:** Characteristics of the study population.

	Total (N = 69)	Early (n = 39)	Late (n = 30)	*p*-Value in Bivariate Analysis
Demographic characteristics				
Age (years) ^a^	35 ± 11 (16–62)	35 ± 10 (16–49)	36 ± 12 (19–62)	0.552
Gender				0.931
Female	28 (41%)	16 (41%)	12 (40%)	
Male	41 (59%)	23 (59%)	18 (60%)	
Injury characteristics				
Side				0.095
Left	40 (58%)	26 (67%)	14 (47%)	
Right	29 (42%)	13 (33%)	16 (53%)	
Meniscus injury (concomitant)	35 (51%)	22 (56%)	13 (43%)	0.281
Lateral	11 (16%)	8 (21%)	3 (10%)	0.327
Medial	25 (36%)	15 (38%)	10 (33%)	0.660
Tegner score before injury	1 (IQR: 1–2)	1 (IQR: 1–2)	1 (IQR: 0–2)	0.306 ^b^
Surgery characteristics				
ACL surgery				0.086
Hamstrings Double Bundle	38 (55%)	25 (64%)	13 (43%)	
Patella BTB	31 (45%)	14 (36%)	17 (57%)	
Meniscus surgery (concomitant)				0.489
None	34 (49%)	17 (44%)	17 (57%)	
Partial resection/debridement	24 (35%)	16 (41%)	8 (27%)	
Repair	11 (16%)	6 (15%)	5 (17%)	
Surgery duration (minutes) ^a^	73 ± 18 (40–117)	74 ± 19 (40–117)	71 ± 17 (42–106)	0.482
Inpatient stay (days) ^a^	3 (IQR: 3–4)	4 (IQR: 3–4)	3 (IQR: 3–4)	0.791 ^b^
Postoperative characteristics				
Follow-up examination (years)	2.8 (IQR: 2.2–3.3)	2.5 (IQR: 2.2–3.5)	2.9 (IQR: 2.4–3.2)	0.321 ^b^
KT 1000 translation				
Absolute values ^a^	5.5 ± 2.2 (2–11)	5.5 ± 2.3 (2–11)	5.4 ± 2.2 (2–11)	0.921
Relative to contralateral leg ^a^	1.0 ± 2.1 (−4–7)	1.1 ± 1.9 (−3–7)	0.93 ± 2.4 (−4–7)	0.827
Tegner score after surgery ^a^	5.5 ± 1.7 (3–10)	5.9 ± 1.6 (3–10)	5.0 ± 1.7 (3–8)	**0.027**
IKDC	73 ± 19 (15–100)	77 ± 16 (36–100)	68 ± 21 (15–100)	**0.045**

Significant differences are in bold type. ^a^ Values are expressed as mean ± SD, with the range in parentheses. ^b^ Two-sample Wilcoxon rank-sum test.

**Table 2:** Concomitant meniscus management.

	Total (n = 35)	Partial Meniscectomy (n = 24)	Repair (n = 11)	*p*-Value in Bivariate Analysis
Classification				>0.999 ^a^
Radial tear	5 (14%)	3 (13%)	2 (18%)	
Flap tear	13 (37%)	9 (38%)	4 (36%)	
Horizontal tear	17 (49%)	12 (50%)	5 (45%)	

^a^ Fisher’s exact test.

**Table 3:** Hierarchical regression analysis for variables predicting postoperative IKDC scores (R^2^ = 0.25).

Variable						Step			
	ß	(SE)	t	*p*-Value	95% CI	Partial R^2^	ΔR^2^	ΔF (df)	*p*-Value
Step 1 (demographics)							0.14	5.4 (2, 66)	**0.007**
Age (years)	−0.36	(0.20)	−1.8	0.074	[−0.75; 0.04]	0.05			
Tegner (preoperative)	4.4	(1.7)	2.6	**0.013**	[0.97; 7.9]	0.09			
Step 2 (time of intervention)							0.04	3.3 (1, 65)	0.075
Age (years)	−0.33	(0.19)	−1.7	0.089	[−0.72; 0.05]	0.04			
Tegner (preoperative)	4.0	(1.7)	2.4	**0.021**	[0.64; 7.5]	0.08			
Early intervention	7.8	(4.3)	1.8	0.075	[−0.82; 16]	0.05			
Step 3 (surgery specifics)							0.01	0.32 (2, 63)	0.725
Age (years)	−0.38	(0.20)	−1.8	0.070	[−0.78; 0.03]	0.05			
Tegner (preoperative)	3.9	(1.7)	2.2	**0.029**	[0.41; 7.4]	0.07			
Early intervention	7.2	(4.4)	1.6	0.106	[−1.6; 16]	0.04			
Meniscus surgery (none)									
Partial meniscectomy	3.6	(4.9)	0.74	0.460	[−6.1; 13]	0.01			
Repair	−0.57	(6.2)	−0.09	0.928	[−13; 12]	<0.01			
Step 4 (interaction: time of intervention and meniscus surgery)							0.06	2.4 (2, 61)	0.098
Age (years)	−0.49	(0.21)	−2.4	**0.022**	[−0.91; −0.07]	0.08			
Tegner (preoperative)	3.6	(1.7)	2.1	**0.042**	[0.13; 7.1]	0.07			
Early intervention	1.9	(6.1)	0.32	0.753	[−10; 14]	<0.01			
Meniscus surgery (none)									
Partial meniscectomy	3.6	(7.5)	0.48	0.634	[−11; 18]	<0.01			
Repair	−16	(9.3)	−1.7	0.100	[−6.1; 13]	0.04			
Meniscus × Early intervention (interaction)									
Partial meniscectomy × Early	2.2	(9.8)	0.22	0.827	[−17; 22]	<0.01			
Repair × Early	27	(13)	2.1	**0.037**	[1.6; 52]	0.07			
Constant (full model)	82	(9.9)	8.3	0.000	[62; 102]				

## Data Availability

Data are available on request from the corresponding author.
